# Bioactivity of the *Cymbopogon citratus* (Poaceae) essential oil and its terpenoid constituents on the predatory bug, *Podisus nigrispinus* (Heteroptera: Pentatomidae)

**DOI:** 10.1038/s41598-019-44709-y

**Published:** 2019-06-07

**Authors:** Bruno Pandelo Brügger, Luis Carlos Martínez, Angelica Plata-Rueda, Barbara Monteiro de Castro e Castro, Marcus Alvarenga Soares, Carlos Frederico Wilcken, Amélia Guimarães Carvalho, José Eduardo Serrão, José Cola Zanuncio

**Affiliations:** 1 Departamento de Entomologia/BIOAGRO, Universidade Federal de Viçosa, 36.570-900 Viçosa, Minas Gerais Brazil; 2 Departamento de Biologia Geral, Universidade Federal de Viçosa, 36.570-900 Viçosa, Minas Gerais Brazil; 3 Programa de Pós-Graduação em Produção Vegetal, Universidade Federal dos Vales do Jequitinhonha e Mucuri (UFVJM), 39100-000 Diamantina, Minas Gerais Brazil; 4 Departamento de Proteção Vegetal, Faculdade de Ciências Agronômicas, Universidade Estadual Paulista (UNESP), 18610-034, Campus de Botucatu, São Paulo, Brazil; 5 Departamento de Engenharia Florestal, Universidade Federal de Viçosa, 36570-900 Viçosa, Minas Gerais Brazil

**Keywords:** Behavioural ecology, Entomology

## Abstract

*Podisus nigrispinus* Dallas (Heteroptera: Pentatomidae), released in biological control programs, is a predator of Lepidopteran and Coleopteran species. Lemongrass essential oil and its constituents can be toxic to this natural enemy. The major constituents of lemongrass essential oil are neral (31.5%), citral (26.1%), and geranyl acetate (2.27%). Six concentrations of lemongrass essential oil and of its citral and geranyl acetate constituents were applied to the thorax of *P*. *nigrispinus* nymphs and adults. The walking and respiratory behavior of the *P*. *nigrispinus* third-instar nymphs, treated with citral and geranyl acetate at the LD_50_ and LD_90_ doses, were analyzed with video and respirometer. The lemongrass essential oil toxicity increased from first- to fifth-instar *P*. *nigrispinus* nymphs. The *P*. *nigrispinus* respiration rates (μL de CO_2_ h^−1^/insect) with citral and geranyl acetate in the LD_50_ and LD_90_ differed. Nymphs exposed to the lemongrass essential oil and its constituents on treated surfaces presented irritability or were repelled. *Podisus nigrispinus* adults were tolerant to the lemongrass essential oil and its constituents, geranyl acetate and citral. The altered respiratory activity with geranyl acetate and the fact that they were irritated and repelled by citral suggest caution with regard to the use of the lemongrass essential oil and its constituents in integrated pest management incorporating this predator, in order to avoid diminishing its efficiency against the pests.

## Introduction

Predatory insects play an important role in insect communities, and are used in biological control to reduce herbivorous arthropod populations^1,2^. The predatory bug, *Podisus nigrispinus* Dallas (Heteroptera: Pentatomidae) can control Lepidopteran and Coleopteran pest species which prey on agricultural crops and forest plantations in the Americas^3–5^. The biology and ecology of *P*. *nigrispinus*, including its development, morphology^6^, predator-prey interaction^4^, and feeding strategies such as extraoral digestion^1^ have been studied. This insect is reared in the laboratory and released in biological control programs in cotton^7^, soybean^4^, and tomato^8^ crops.

Synthetic insecticides may induce resistance in insects^9^, cause toxic reactions in mammals^10^ and other non-target organisms such as parasitoids, pollinators, and predators^11–13^, and may also leave residues^14^ and cause environmental pollution^15^. Exposure to insecticide causes adverse effects on the development, longevity and fecundity, and may alter behavior related to mobility and feeding^16–18^. The search for safer insecticides for human health and the environment has resulted in the development of specific compounds for pests which are selective for non-target organisms^19,20^. In this sense, effective use of *P*. *nigrispinus* in integrated pest management (IPM) programs depends on the compatibility of the predator with the other control methods being employed^21^.

Plant essential oils represent an alternative for pest control with low pollution and quick degradation in the environment, making them suitable for managing insects even in organic farming^22–24^. Plant essential oils are volatile substances, mainly composite mixtures of terpenoids which are used for their aromatic qualities. In plants, terpenoids are products of secondary metabolism and are found in glandular hairs or secretory cavities of the plant cell wall in bark, flowers, fruits, leaves, roots and stems^25^. Essential oils and their constituents cause lethal and sublethal effects on insects, such as biocide activity, infertility, irritability, phagoinhibition and repellency^23,26,27^. Essential plant oils can control pests^22,23,26,27^.

Lemongrass, *Cymbopogon citratus* (DC. Stapf.), a plant native to India and Sri Lanka^28^, has antifungal^29^, anti-inflammatory^30^ and anti-protozoa^31^ properties. Predatory insects have a tolerance in relation to essential oils, which emphasizes the importance of the potential success of these natural enemies in IPM programs^24^. Essential oils have been shown to possess toxic effects against lepidopteran pests such as *Euprosterna elaeasa* Dyar (Limacodidae)^32^, *Spodoptera exigua* Hübner^33^ and *Trichoplusia ni* Hübner (Noctuidae)^34^, and these insects are natural prey of the *P*. *nigrispinus* in Brazilian agricultural crops. However, the lethal and sublethal effects caused by essential oils have also been demostrated on this predatory bug^35^. *Podisus nigrispinus* is a predator of defoliating pests in different crop systems, but the action of essential oils as insecticide on this natural enemy of those pests needs further studies in order to avoid harming this natural ally.

The objective of this study was to evaluate the lethal and sublethal effects of lemongrass essential oil and its terpenoid constituents (geranyl acetate and citral) on *P*. *nigrispinus*.

## Results

### Lemongrass essential oil toxicity test

Lethal doses of the lemongrass oil increased from first to fifth instars with LD_50_ of 1.08 to 139.30 μg/insect^−1^ and LD_90_ of 2.02 to 192.05 μg/insect^−1^. The LD_50_ and LD_90_ of the lemongrass for third instar *P*. *nigrispinus* nymphs was 21.58 and 28.35 μg/insect^−1^, respectively. Mortality was always <1% in the control (Table 1).

**Table 1:** Lethal doses of the lemongrass essential oil to different *Podisus nigrispinus* (Heteroptera: Pentatomidae) nymph-instars obtained from Probit analysis (DF = 5).

^1^NI	^2^LD	^3^EV (µg insect^−1^)	^4^CI (µg insect^−1^)	^5^χ^2^ (*P*-value)
I	LD_50_ LD_90_	1.0 2.0	0.8–1.2 1.8–2.2	15.1 (0.26)
II	LD_50_ LD_90_	5.0 8.0	4.5–5.4 7.4–8.7	17.0 (0.29)
III	LD_50_ LD_90_	21.5 28.3	20.6–22.5 26.7–30.7	13.3 (0.32)
IV	LD_50_ LD_90_	56.9 64.3	53.5–63.8 53.9–87.7	2.49 (0.89)
V	LD_50_ LD_90_	139.3 192.0	108.1–400.2 138.2–656.7	1.89 (0.91)

The chi-square value refers to the goodness of fit test at *P* > 0.05. ^1^NI, instars; ^2^LD, lethal doses (LD_50_ and LD_90_) corresponding to 50 and 90% mortality; ^3^EV, estimated value; ^4^CI, confidence interval; ^5^χ^2^, chi-square and *P* value for the lethal doses and confidence intervals.

### Composition of lemongrass essential oil

A total of 13 compounds from the lemongrass essential oil were identified, which accounted for 95.98% of its total composition (Table 2). The primary compounds of the lemongrass oil were neral (31.5%), citral (26.1%), nonan-4-ol (6.54%), camphene (5.19%), 6-metil-hept-5-en-2-one (4.36%), citronelal (3.83%), β-caryophyllene (3.26%), citronelol (2.95%), caryophyllene oxide (2.63%), γ-muurolene (2.46%), limonene (2.32%), geranyl acetate (2.27%), and geranial (2.15%).

**Table 2:** Chemical composition of the lemongrass essential oil.

Peaks	Constituents	R_i_	R_t_	MC	MM	*m/z*
1	6-methylhept-5-en-2-one	938	8.91	4.36	128	121.1
2	Camphene	958	10.8	5.19	130	108.1
3	Limonene	1030	12.4	2.32	136	94.1
4	Nonan-4-ol	1052	14.7	6.54	142	86.1
5	Citronellal	1125	18.5	3.83	154	121.1
6	Citronellol	1136	19.8	2.95	156	109.1
7	Neral	1174	22.1	31.5	156	95.1
8	Geraniol	1179	22.5	2.15	152	109.1
9	Citral	1228	23.2	26.1	154	123.1
10	Geranyl acetate	1274	23.8	2.27	196	137.1
11	β-caryophyllene	1352	28.8	3.26	204	136.1
12	γ-muurolene	1435	29.9	2.46	204	133.1
13	Caryophyllene oxide	1494	33.8	2.63	220	204.1

R_i_, retention indices; R_t_, retention time; MC, mean composition (% Area); MM, molecular mass; *m/z*, mass-to-charge ratio.

### Toxicity of lemongrass commercial constituents

**Figure 1:**
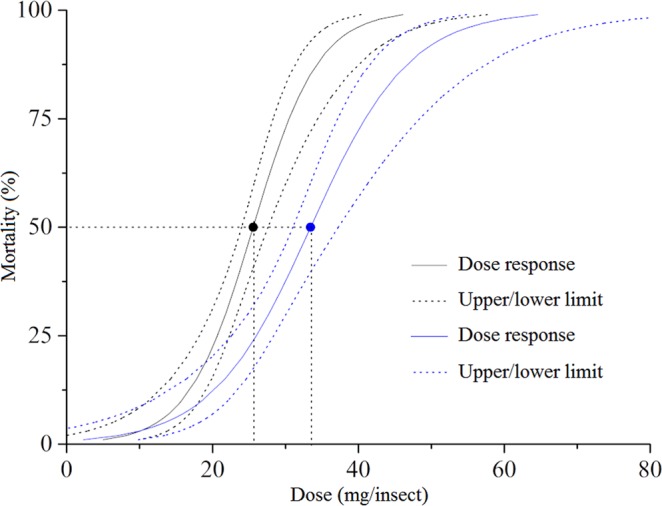
Mortality curve, estimated by the dose-response (Probit), of *Podisus nigrispinus* (Heteroptera: Pentatomidae) nymphs for geranyl acetate and citral at two lethal doses (LD_50_ and LD_90_) (*X*^2^; *P* < 0.001). Dotted lines denote 95% confidence intervals. Black dot represents LD_50_ (citral) and blue LD_50_ (geranyl acetate) selected to assess the toxic effects.

The dose response with geranyl acetate showed that this compound has lower toxicity than citral in third-instar *P*. *nigrispinus* nymphs, with LD_50_ = 33.44 (30.99–37.23) μg/insect^−1^ and LD_90_ = 48.34 (42.78–59.99) μg/insect^−1^, compared to LD_50_ = 25.56 (23.98–27.60) μg/insect^−1^ and LD_90_ = 35.39 (32.15–41.61) μg/insect^−1^ for the citral (Fig. 1).

### Effects on the respiratory rate

**Figure 2:**
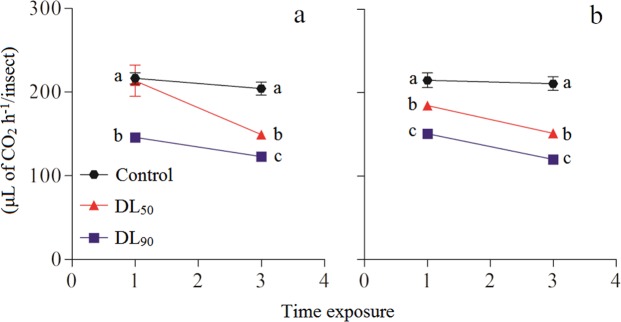
Respiration rate (Mean ± SD) of *Podisus nigrispinus* (Heteroptera: Pentatomidae) after exposure to geranyl acetate and citral at the LD_50_ and LD_90_ doses on third-instar nymphs: geranyl acetate (**a**), citral (**b**). Treatments (Mean ± SD) differ in *P* < 0.05 (Tukey mean separation test).

The respiration rate (μL de CO_2_ h^−1^/insect) of third-instar *P*. *nigrispinus* nymphs differed between the LD_50_ and LD_90_ of geranyl acetate (F_2,48_ = 4.81; *P* < 0.001) (Fig. 2a), and of citral (F_2,48_ = 22.19; *P* < 0.001) (Fig. 2b). The respiratory rate for third-instar *P*. *nigrispinus* nymphs differed between 1 and 3 h of exposure to geranyl acetate (F_2,48_ = 5.12, *P* < 0.001) or citral (F_2,48_ = 8.32; *P* < 0.001).

### Effects on locomotor behavior

**Figure 3:**
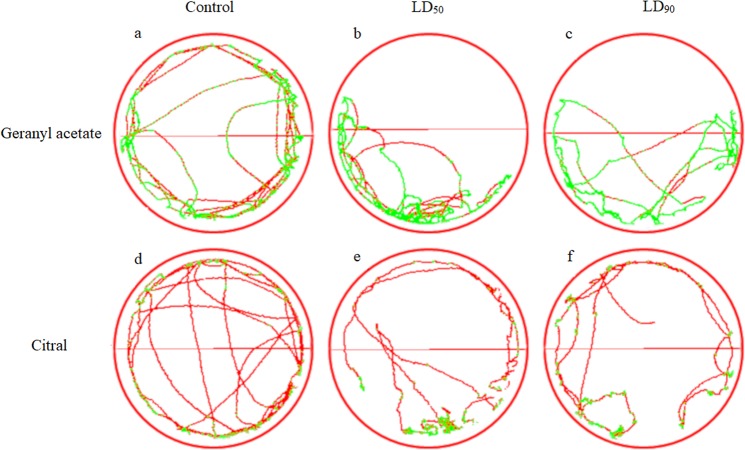
Representative locomotor activity tracks of *Podisus nigrispinus* (Heteroptera: Pentatomidae) nymphs for 10 minutes in filter paper arrays (9 cm in diameter) impregnated in the upper half of each arena with geranyl acetate (**a**: Control, **b**: LD50 and **c**: LD90) or citral (**d**: Control, **e**: LD_50_ and **f**: LD_90_). Red tracks indicate high-speed walking and green indicates low speed (initial).

**Figure 4:**
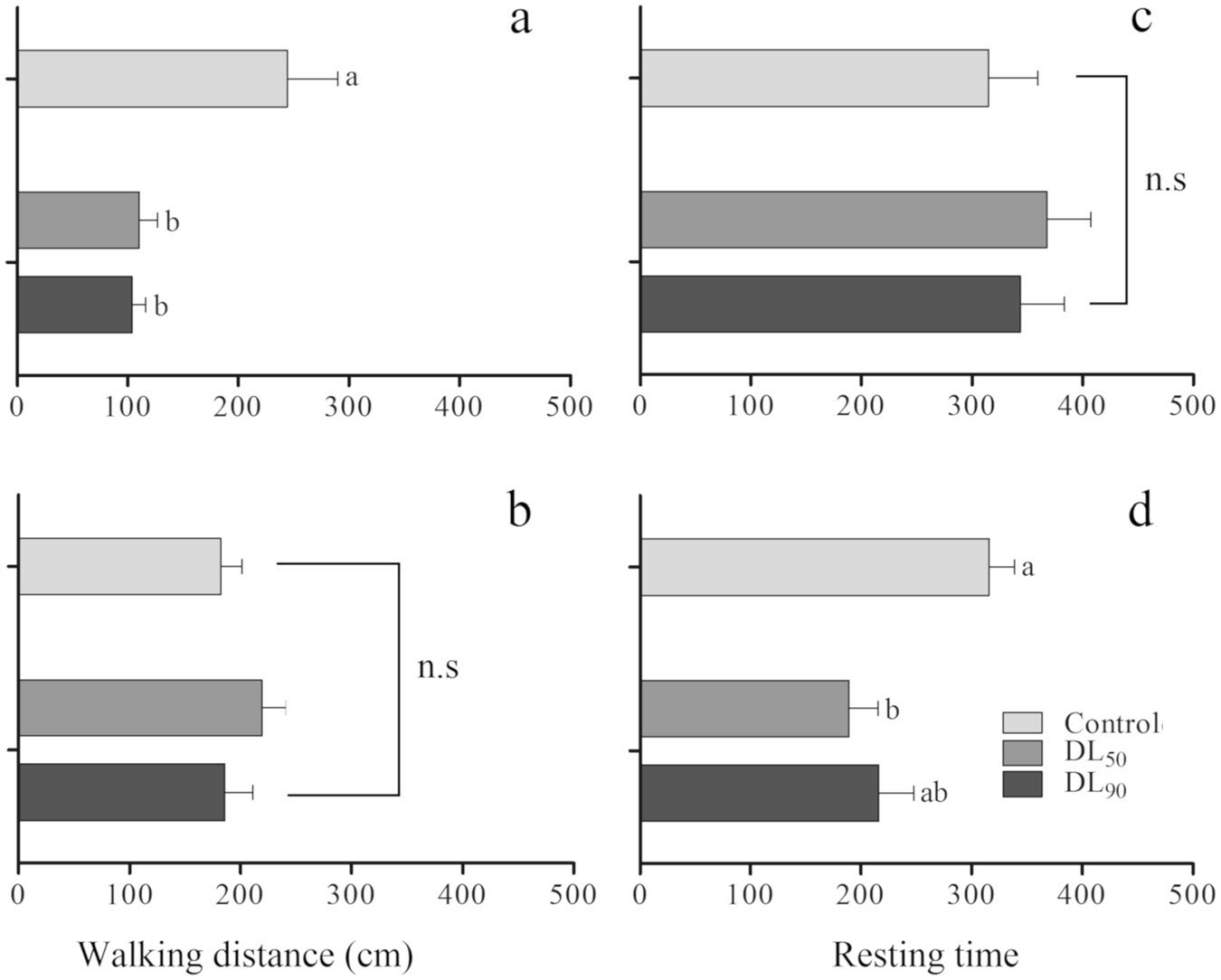
Walking distance (**a**,**b**) and resting time (**c**,**d**) (Mean ± SD) of third instar *Podisus nigrispinus* (Heteroptera: Pentatomidae) nymphs with LD_50_ and LD_90_. Treatments (Mean ± SD) differ in *P* < 0.05 (Tukey mean separation test). Geranyl acetate (**a**,**c**) and citral (**b**,**d**).

The representative bands of the *P*. *nigrispinus* trail in the treated arena part indicated that the geranyl acetate repelled and by citral irritated this predator(Fig. 3). The walking distance with geranyl acetate was shorter (F_2,11_ = 8.21, *P* < 0.002) (Fig. 4a) and the immobile period longer (F_2,11_ = 0.41, *P* = 0.669), (Fig. 4c). The walking distance of *P*. *nigrispinus* exposed to citral did not differ from the control (F_2,11_ = 0.83, *P* = 0.449) (Fig. 4b), but the immobile period of this predator (F_2,11_ = 4.99, *P* < 0.016) was longer with this compound (Fig. 4d).

## Discussion

The increase in the lethal doses (LD_50_ and LD_90_) of the lemongrass essential oil from first to fifth instars of *P*. *nigrispinus* suggests that this predator progressively developed a tolerance as it matured. The toxicity of this essential oil is similar to that reported in other studies for this insect^36^ and for other predators^37,38^, showing a relatively favorable safety profile for *P*. *nigrispinus*, whether through direct use of the oil or by the individual components of the oil serving as precursors for the synthesis of active ingredients of new selective insecticides. Insects may present selectivity mechanisms such as reduction of insecticide penetration through the cuticle, or site insensitivity and/or detoxification or metabolization of the insecticide by enzymes^39^ to reduce the effect on acetylcholinesterase^40^ or inhibition of octopamine receptors^41^. Comparing the contact toxicity of lemongrass essential oil on developmental of *P*. *nigrispinus* nymphs showed that the first and second instar were more susceptible followed by the third, fourth and fifth instars; this indicates that high quantities of the lemongrass essential oil are toxic in the early stages of this insect, and that they become more tolerant with age.

The chemical composition of lemongrass essential oil revealed 13 constituents, identified and quantified. Neral, citral, nonan-4-ol, camphene, 6-metil-hept-5-en-2-one, and citronelal were the main compounds that were detected, according to previous reports on terpenoids obtained from lemongrass essential oil^42–44^. However, variations in the abundance of the constituents was observed, including geranial as its main compound^42–44^, depending on the extracted organ, plant age, geographical area of the collection and extraction method^45,46^. Terpenoids are frequently found in plants, where they play numerous vital roles in plant physiology as well as important functions in all cellular membranes^47^. Also, the defensive role in plants containing simple terpenoids has been demonstrated, as well as more complex compounds^48^. In this study, terpenoids are the most abundant constituents of lemongrass essential oil, but the relative proportions of the constituents with insecticide potential^45^ can vary.

The low toxicity of citral or geranyl acetate for *P*. *nigrispinus* may be related to the cuticle of this insect, which acts as a barrier, as reported for *Bombyx mori* Linnaeus (Lepidoptera: Bombycidae) exposed to deltamethrin^49^. Cuticular lipids prevent the desiccation and penetration of xenobiotics into insects^50^, as well as promoting thickening and cuticle composition that can delay the penetration of insecticidal molecules into the body of the insect^51^, thus reducing the essential oil effect post-application due to rapid degradation or evaporation in the environment^52^. The lack of detoxifying enzymatic activity (inhibitors of cytochrome P450s, esterases or glutathione S-transferases)^53^ was observed in *Trichoplusia ni* (Hübner) (Lepidoptera: Noctuidae) treated with citral, which penetrated through the cuticular layer^54^. Toxic constituents can affect multiple regions of the insect body, causing necrotic areas which increase progressively throughout the entire insect body^26,55^. One possible explanation for the low toxicity caused in *P*. *nigrispinus* is that there may be differences in the penetration rate of the lemongrass constituents into the body, coupled with the ability of this insect to rapidly detoxify.

The terpenoid constituents of lemongrass essential oil had a negative effect on the *P*. *nigrispinus* respiration rate. The reduction of the respiratory rate of third-instar *P*. *nigrispinus* nymphs after exposure to citral and geranyl acetate may be due to muscle paralysis, disruption of oxidative phosphorylation processes and dysregulation of the breathing activities^18,22,26,56^. In this study, *P*. *nigrispinus* nymphs exposed to the terpenoid constituents of the essential oil developed low respiration rates, which further unbalanced the organism physiology, as described for *Sitophilus granarius* Linnaeus (Coleoptera: Curculionidae)^57^ and *Tenebrio molitor* Linnaeus (Coleoptera: Tenebrionidae)^55^.

The short distance traveled in the arenas towards the opposite side from the geranyl acetate by *P*. *nigrispinus* nymphs suggest repellent activity. Various insect pests show altered behavioral responses when exposed to lemongrass essential oil constituents, as reported for *Bemisia tabaci* Gennadius (Hemiptera: Aleyrodidae)^58^, *Culex quinquefasciatus* Say (Diptera: Culicidae)^59^, and *Tribolium castaneum* (Herbst) (Coleoptera: Tenebrionidae)^60^, influencing the olfactory orientation and insect walking behavior. Insects can identify the presence of compounds, as reported for *Acyrthosiphon pisum* (Harris) (Hemiptera: Aphididae), after the activation of olfactory receptors in the presence of geranyl acetate^61^. The results indicate that *P*. *nigrispinus* exhibits behavioral avoidance by means of repellence to geranyl acetate, minimizing contact with insecticide-contaminated surfaces. In contrast, nymphs of *P*. *nigrispinus*, exposed in arenas with citral, presented irritability and decreased resting periods, which may be related to intoxication in the octapaminergic system, causing hyperactivity or hyperextension in the legs and abdomen^62^. Essential oils have caused sublethal effects such as increased heart rate, changes in the cAMP level in the nervous system and decreased binding to octopamine receptors, as decribed for *Periplaneta americana* Linnaeus (Blattodea: Blattidae)^62^.

*Podisus nigrispinus* tolerates lemongrass essential oil and its constituents, but geranyl acetate repelled this predator and citral caused irritability. This suggests caution in the use of lemongrass essential oil and these constituents in integrated pest management involving *P*. *nigrispinus*. This study may support future research with *Cymbopogon citratus* and its constituents in the search for bioinsecticides, based on nanoscience, against pests but without effect on this predator.

## Methods

### Insect mass rearing

Nymphs and adults of *P*. *nigrispinus* were obtained from the mass rearing of the ‘Laboratorio de Controle Biológico’ (LCBI) of the ‘Universidade Federal de Viçosa’ (UFV) in Viçosa, Minas Gerais state, Brazil. This predatory bug is reared at room temperature at 25 ± 1 °C, 70 ± 10% RH, and 12 h photophase. *Podisus nigrispinus* eggs were placed in Petri dishes (12 × 1.5 cm) with cotton soaked with water. Nymphs and adults of this insect were monitored in cubic wooden cages (30 × 30 × 30 cm) covered with nylon. These nymphs and adults were fed *ad libitum* with *Tenebrio molitor* L. (Coleoptera: Tenebrionidae) pupae and received *Eucalyptus* sp. (Myrtaceae) leaves and water^2^.

### Essential oil toxicity test

The lemongrass essential oil was acquired from the ‘Destilaria Bauru Ltda.’ company (Catanduva, São Paulo, Brazil), extracted by hydrodistillation on an industrial scale^63^. Lemongrass essential oil was diluted in 1 mL of acetone to obtain a stock solution. Six different doses of lemongrass were prepared and used to assess the insecticide toxicity and determine relevant toxicological endpoints; a dilution series of doses (8.1, 16.2, 31.2, 62.5, 125, and 250 µg/insect^−1^) was used to determine dose-mortality relationship and lethal dose (LD_50_ and LD_90_). Acetone was used as a control. Each solution (1 μL) was applied to the thorax of first-, second-, third-, fourth- and fifth-instar nymphs using a micropipette. For each nymph instar, fiften nymphs were tested, placed individually in Petri dishes with one *T*. *molitor* pupa per day and cotton soaked with water. The number of dead nymphs in each Petri dish was counted after 36 h.

### Identification of the lemongrass essential oil constituents

Quantitative analyses of lemongrass essential oil were performed in triplicate using a gas chromatograph (GC-17A, Shimadzu, Kyoto, Japan) equipped with flame ionization detector (FID). Chromatographic conditions were: a fused silica capillary column (30 m × 0.22 mm) with a DB-5 bonded phase (0.25 μm film thickness); carrier gas N_2_ at a flow rate of 1.8 mL min^−1^; injector temperature of 220 °C; detector temperature of 240 °C; column temperature programmed to begin at 40 °C (remaining isothermal for 2 min) and increase at 3 °C min^−1^ to 240 °C (remaining isothermal at 240 °C for 15 min); 1 μL injection volume (1% w/v in dichloromethane); 1:10 split ratio and 115 kPa column pressure.

Constituents were identified using a gas chromatograph coupled with a mass detector GC/MS (CGMS-QP 5050 A; Shimadzu, Kyoto, Japan). The injector and detector temperatures were 220 °C and 300 °C, respectively. The initial column temperature was 40 °C for 3 min, with a programmed temperature increasing of 3 °C/min to 300 °C, where it was maintained for 25 min. The split mode ratio was 1:10. One microliter of lemongrass essential oil containing 1% (w/v in dichloromethane) was injected and helium used as carrier gas with a flow rate constant of 1.8 mL^−1^ on the Rtx^®^-5MS capillary column (30 m, 0.25 mm × 0.25 μm, Bellefonte, USA) using Crossbond® stationary phase (35% diphenyl, 65% dimethyl polysiloxane). The Mass Spectrometer was programmed to detect masses in the range of 29–450 DA with 70 eV ionization energy. Constituents were identified by comparisions of the mass spectra with those available from the National Institute of Standards and Technology (NIST08, NIST11) libraries, the Wiley Spectroteca database (7^th^ edition), and by the retention indices.

### Toxicity of lemongrass constituents

Geranyl acetate (97.0% purity) and citral (95.0% purity), identified as constituents of the lemongrass essential oil, were obtained from Sigma Aldrich (Darmstadt, Germany). The efficacy of these constituents was determined by their lethal doses (LD_50_ and LD_90_) in the laboratory. Six different doses of each constituent were prepared and used to assess the insecticide toxicity and determine relevant toxicological endpoints; a dilution series of doses (8.1, 16.2, 31.2, 62.5, 125, and 250 µg/insect^−1^) was used to determine dose-mortality relationship and lethal dose. Acetone was used as a control. Each solution (1 μL) was applied to the thorax of third-instar nymphs using a micropipette. Fiften nymphs were tested, placed individually in Petri dishes with one *T*. *molitor* pupa per day, and cotton soaked with water. The number of dead nymphs in each Petri dish was counted after 36 h.

### Testing the respiratory rate

Respiration rate bioassays were conducted for 3 h after *P*. *nigrispinus* nymphs were exposed to geranyl acetate or and citral (LD_50_ and LD_90_ levels). Insects treated with distilled water were used as control. Carbon dioxide (CO_2_) production (μL of CO_2_ h^−1^/insect) was measured with a TR3C CO_2_ Analyzer (Sable System International, Las Vegas, USA) according to methods adapted from previous studies^18,22,55^. A third-instar nymph of *P*. *nigrispinus* was placed in each respirometry chamber (25 mL) connected to a closed system. After insect acclimation, CO_2_ production was measured for 12 h at 27 ± 2 °C. Subsequently, compressed oxygen gas (99.99% pure) was introduced into the chamber at 100 mL min^−1^ for 2 min. The gas flow forces the CO_2_ through an infrared reader, which continuously measures the CO_2_ contained inside the chamber. Before and after the experiment, *P*. *nigrispinus* nymphs were weighed on an analytical balance (Sartorius BP 210D, Göttingen, Germany). Ten replicates were used for each insecticide treatment and control following a completely randomized design.

### Testing locomotion behavior

Nymphs of *P*. *nigrispinus* were placed in a Petri dish (90 mm diameter × 15 mm high) lined with filter paper (Whatman no. 1). Then the inner walls of the Petri dish were covered with polytetrafluoroethylene (Dupont^®^, Barueri, SP, Brazil) to prevent insect escape. Behavioral locomotor response bioassays were conducted in arenas half-treated with 250 µL of geranyl acetate or citral; dishes treated with acetone only were used as control. One *P*. *nigirispinus* nymph was released at the center of the arena treated with geranyl acetate or citral (on filter paper) and kept in the Petri dish for 10 min. Forty-eight third-instar *P*. *nigrispinus* nymphs were used for each lethal dose (16 per each treatment: control, geranyl acetate or citral), following a completely randomized design. For each insect, walking activity within the arena was recorded using a digital camcorder (XL1 3CCD NTSC, Canon, Lake Success, NY, USA) equipped with a 16 × video lens (Zoom XL 5.5–88 mm, Canon, Lake Success, NY, USA). A video tracking system (ViewPoint LifeSciences, Montreal, Quebec, Canada) was used to analyze the videos and measure the distances that the insects walked and the time spent resting on each half of the arena. Insects that spent less than 1 s on the half of the arena treated with the essential oil or constituent were considered repelled, whereas those that remained less than 50% of the time on the insecticide-treated surface were considered to have been irritated^26,55,57^.

### Statistical analysis

Dose-mortality data were subjected to Probit analysis, generating a dose-mortality curve^64^. Respiration rates were subjected to two-way ANOVA and Tukey’s HSD test (*P* < 0.05). Locomotor behavior response data were analyzed by one-way ANOVA, and a Tukey Honestly Significant Difference (HSD) test was also used for comparison of means at the 5% significance level. Toxicity, respiration rate, and locomotor behavior response data were analyzed using SAS for Windows v. 9.0^65^.
